# Level of ERAS understanding affects practitioners’ practice and perception of early postoperative resumption of oral intake: a nationwide survey

**DOI:** 10.1186/s12871-021-01500-9

**Published:** 2021-11-12

**Authors:** Huizhen Huang, Yuelun Zhang, Le Shen, Yuguang Huang

**Affiliations:** 1 Department of Anesthesiology, Peking Union Medical College Hospital, Beijing, 100730 China; 2 Medical Research Center, Peking Union Medical College Hospital, Beijing, 100730 China

**Keywords:** Postoperative, Oral intake, Enhanced recovery after surgery, Survey

## Abstract

**Background:**

Early postoperative resumption of oral intake is supposed to be safe and beneficial to patients recovery. However, practitioners still have great confusion and disagreement about postoperative resumption of oral intake. This is a nationwide survey to investigate the current status of clinical practice and practitioners’ attitude toward postoperative resumption of oral intake along with their level of understanding of the ERAS guidelines.

**Methods:**

An anonymous web-based survey questionnaire via mobile social platform was carried out in mainland China from December 11–20, 2020. The Wilcoxon signed rank test or chi-square test was used to compare the propensity of the resumption of oral intake.

**Results:**

Totally 5370 responses were received, and 89% of them were from anesthesiology departments. The nature of the responses from clinical practitioners was highly diverse, but each of the three surgery types showed unique patterns of ERAS implementation. The respondents were more conservative regarding the commencement of both fluid and solid diets after gastrointestinal (GI) and hepato-pancreato-biliary (HPB) surgery than after non-abdominal (NA) surgery. Most respondents agreed that early oral intake is beneficial to reduce postoperative complications improve bowel recovery and overall outcome. 55% respondents considered themselves to have a better understanding of ERAS and tended to initiate oral intake early for all three surgery types (*P* < 0.001).

**Conclusions:**

The postoperative resumption of oral intake is highly variable among GI, HPB and NA surgeries. A better understanding of ERAS would encourage practitioners to commence oral intake resumption much earlier.

**Supplementary Information:**

The online version contains supplementary material available at 10.1186/s12871-021-01500-9.

## Background

Traditionally, postoperative oral intake is gradually introduced following the resumption of bowel sounds and the passage of flatus or stool, which is mainly due to the fear that early oral intake can prolong paralytic ileus. However, a growing body of evidence has suggested these fears are ungrounded, and it has been widely accepted that any delay in the resumption of a normal oral diet after major surgery is associated with increased rates of surgical site infection and delayed recovery [1–3]. As it is safe and beneficial to patients, the concept of early postoperative oral intake has been increasingly incorporated into enhanced recovery after surgery (ERAS) protocols [4, 5]. ERAS clinical pathways have been developed with the goal of improving the quality of perioperative care, minimizing the loss of functional capacity and enhancing the recovery process. The ERAS program initially became well established for colorectal surgery [6] and has been further expanded, promoting rapid functional recovery after abdominal and non-abdominal surgeries such as gastrointestinal surgery [7], pancreatoduodenectomy [8], liver surgery [9], lung surgery [10], breast surgery [11], total hip/knee replacement [12], lumbar spinal fusion [13], cardiac surgery [14] and gynecological surgeries [15, 16].

Almost all these ERAS programs suggest re-establishing oral feeding as early as possible after surgery. Recently, the European Society for Clinical Nutrition and Metabolism (ESPEN) nutrition guidelines [17] have recommended that in general, oral nutritional intake should be continued after surgery without interruption and that oral intake including clear liquids should be initiated within hours after surgery in most patients. (Evidence level: I, Recommendation grade: A). However, considering the original bowel function state and the impact of surgery is variable among patients with different conditions, the guidelines also noted that oral intake should be adapted according to individual tolerance and to the type of surgery carried out, with special caution taken for elderly patients. It deserves due attention that unlike the “2–4–6–8 rule” as a universally accepted standard of preoperative fasting [18], when to commence postoperative oral intake is vague and confusing.

On the other hand, despite the clinical benefits of early resumption of oral intake, its implementation in clinical practice has been relatively “delayed”. Possible reasons include a lack of convincing data, a low-level understanding of ERAS, and limitations in institutional experience and systems [19]. Therefore, in an effort to identify difficulties in the implementation of early postoperative oral intake programs, we carried out a nationwide survey to determine clinical practices and attitudes toward the postoperative resumption of oral intake among all clinical practitioners along with their level of understanding of ERAS.

## Methods

Ethics: Ethical approval for this study (Ethical Committee No. S-K984) was provided by the Ethical Committee of Peking Union Medical College Hospital, Beijing, China (Chairperson Prof Zhaohui Zhu) on December 11, 2019. As the participation was voluntary and no patients were involved, the Ethical Committee approved this study waived the need for written informed consent.

The “Checklist for Reporting the Results of Internet E-Surveys” (CHERRIES) statement was used for this web-based survey study to improve the quality of reports [20]. We composed an anonymous electronic survey using an online survey tool (https://www.wenjuan.com/). The questionnaire of this survey consisted of 18 single choice, multiple choice and five-point Likert scale questions. If necessary, questions were given an additional free-text option for comments. The survey was piloted and further refined based on initial feedback. The survey questions and structure are shown in Additional file 1. The survey was distributed via a widely used social platform App called WeChat. Two WeChat group chats including all the National Committee Members of Chinese Society of Anesthesiology (CSA) and all the Presidents of Provincial Society of Anesthesiology of China respectively were used for the primary distribution of the survey. And the survey was secondarily distributed in each Provincial Society of Anesthesiology WeChat group chat that included Regional Committee Members of Provincial Society of Anesthesiology. Furthermore, the survey was distributed by each National or Regional Committee Members to anesthesiologists, surgeons and nurses in her or his institution. Each WeChat registered user was allowed to submit the survey questionnaire only once to prevent duplicates. The survey was live from December 11–20, 2020.

Since each respondent gave answers on same questions, when we compared those answers, we considered those data were paired: a set of answers was related to one respondent. We compared the data between different categories of surgeries and between clinical practices and clinician perceptions using the Wilcoxon signed rank test. We compared the data from respondents that had a good understanding of the ERAS with the data from respondents who had less understanding of the ERAS using the chi-square test. All data were analyzed with SPSS [version 20 (SPSS, Chicago, IL, USA)], and two-sided *p* values < 0.05 were considered statistically significant.

## Results

**Fig. 1:**
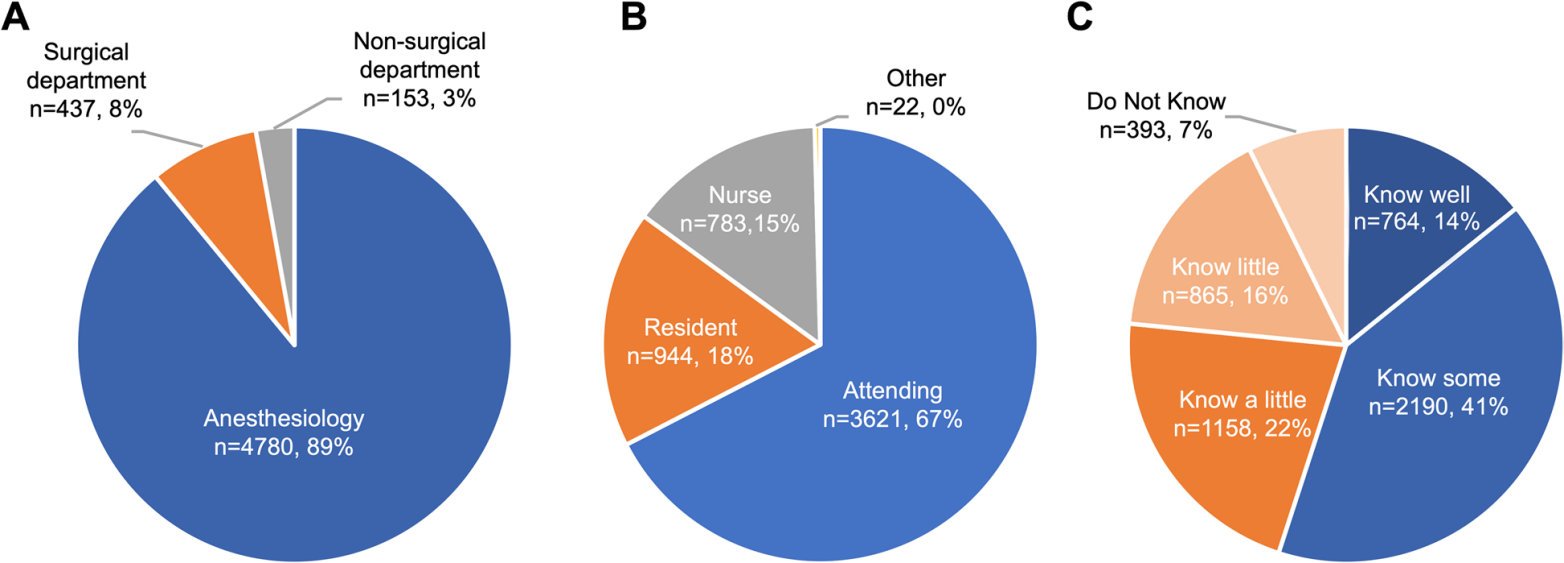
Respondent characteristics, including departments (**A**), titles (**B**) and level of understanding about ERAS (**C**)

There were 13,191 WeChat registered users who opened the web link of this survey. A total of 5370 completed questionnaires were collected, the average response time was 20 min, and the completion rate was 40.71%. The major characteristics of the respondents are shown in Fig. 1. Most of the respondents were from anesthesiology departments (89%), followed by surgical departments (8%) and nonsurgical departments (3%). Eighty-five percent of the respondents were doctors, including 3621 attendings and 944 residents. Nurses were also invited to complete this survey, accounting for 15% of all responses. The respondents were asked to take a self-assessment on their degree of understanding of ERAS in the form of a question based on a five-point Likert scale (know well/know some/know a little/know little/do not know). Most claimed that they “know some” (41%), while 7% reported “do not know”. Based on their understanding of ERAS, respondents were subdivided into two groups: better understanding of ERAS group (know well & know some) (*n* = 2954, 55%) and less understanding of ERAS group (know a little, know little and do not know) (*n* = 2416, 45%) (Fig. 1C).

### Postoperative oral intake in practice and perception

**Fig. 2:**
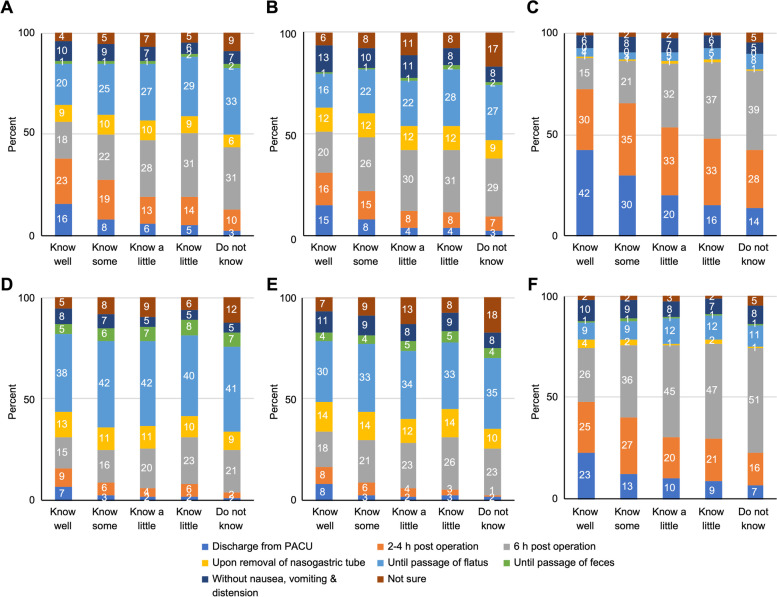
Overall responses for the postoperative resumption of oral intake in clinical practice along with respondents’ level of understanding of ERAS. Resumption of oral fluids after (**A**) gastrointestinal surgery, (**B**) hepato-pancreato-biliary surgery, and (**C**) non-abdominal surgery, and resumption of solid diet after (**D**) gastrointestinal surgery, (**E**) hepato-pancreato-biliary surgery, and (**F**) non-abdominal surgery. PACU, postanesthesia care unit. Data presented as % in columns

Figure 2 shows an overview of the commencement of postoperative oral intake. The nature of the responses from the clinical practitioners was highly diverse, while each of three surgeries showed unique patterns. Respondents took a more conservative approach for gastrointestinal and hepato-pancreato-biliary surgery, in which almost half (at least 47%) allowed patients to begin oral intake “6 hours postoperation” “upon removal of nasogastric tube” or “until passage of flatus”. While compared to the other two surgeries, non-abdominal surgery showed more concentrated patterns in the resumption of oral liquids and solid diet. For non-abdominal surgery, most physicians allowed oral fluids after “discharge from postanesthesia care unit (PACU)” or just “2-4 hours post operation”. Regardless of which surgery was performed, respondents asked patients to refrain from eating longer than drinking. Moreover, although the majority of responses regarding the postoperative resumption of oral fluids in gastrointestinal and hepato-pancreato-biliary surgery were not fixed, the respondents showed a surprising amount of consensus in regard to the resumption of a solid diet—namely, “after the passage of flatus” (in each column, > 38% in the former group and > 30% in the latter group).

When combined with the horizontal axis—understanding of ERAS—the color strips and numbers (representing the percentage of respondents in each group) clearly indicate that respondents that consider themselves to have a better understanding of ERAS tended to commence oral intake early. A close look at the bottom half of figures revealed that clinical practitioners who considered themselves more familiar with ERAS were more likely to commence oral intake once discharged from the PACU or “2-4 hours postoperation”, rather than “6 hours postoperation”. On the other hand, respondents who considered themselves less familiar with ERAS (“do not know”) reported more difficulty in deciding when to commence of postoperative oral intake (“not sure”), regardless of whether oral fluids or a solid diet was being considered.

**Fig. 3:**
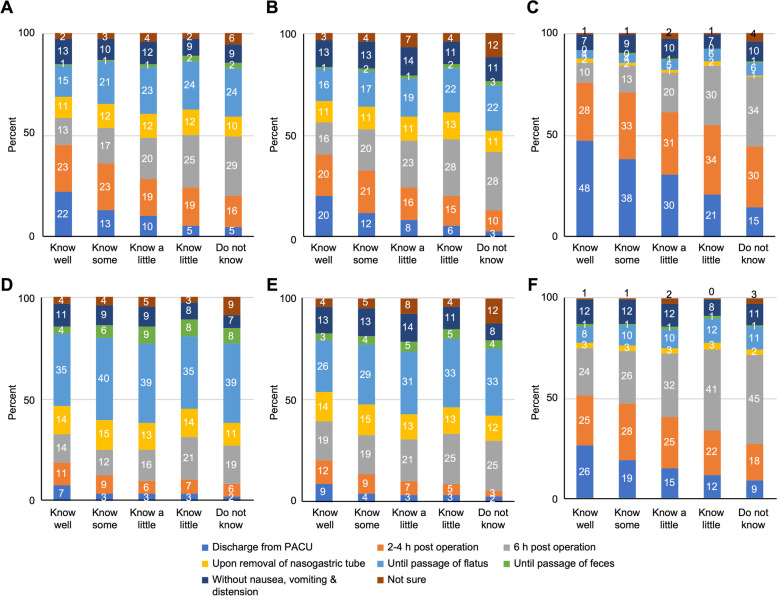
Overall perception of the postoperative resumption of oral intake along with the respondents’ understanding of ERAS. Resumption of oral fluids in (**A**) gastrointestinal surgery, (**B**) hepato-pancreato-biliary surgery, and (**C**) non-abdominal surgery and solid diet in (**D**) gastrointestinal surgery, (**E**) hepato-pancreato-biliary surgery, and (**F**) non-abdominal surgery. PACU, postanesthesia care unit. Data presented as % in columns

Figure 3 shows an overview of the postoperative resumption of oral intake in terms of respondent expectations. The patterns between clinical practice and perception were comparable, and similar to practice, there was a strong tendency for respondents who knew less about ERAS to appear more conservative in their expectations. Overall though, respondents’ expectations were more aggressive than their practice. It is very interesting that clinicians who responded that they “know well” ERAS had the minimum relative difference between practice and perception, exhibiting the most confidence in their decision regarding early resumption of oral intake. Among all the options, the fewest number of respondents waited to resume postoperative oral intake “until the passage of feces” in both practice and perception, regardless of surgery type.

### Surgical and perception differences in the early postoperative resumption of oral intake

**Fig. 4:**
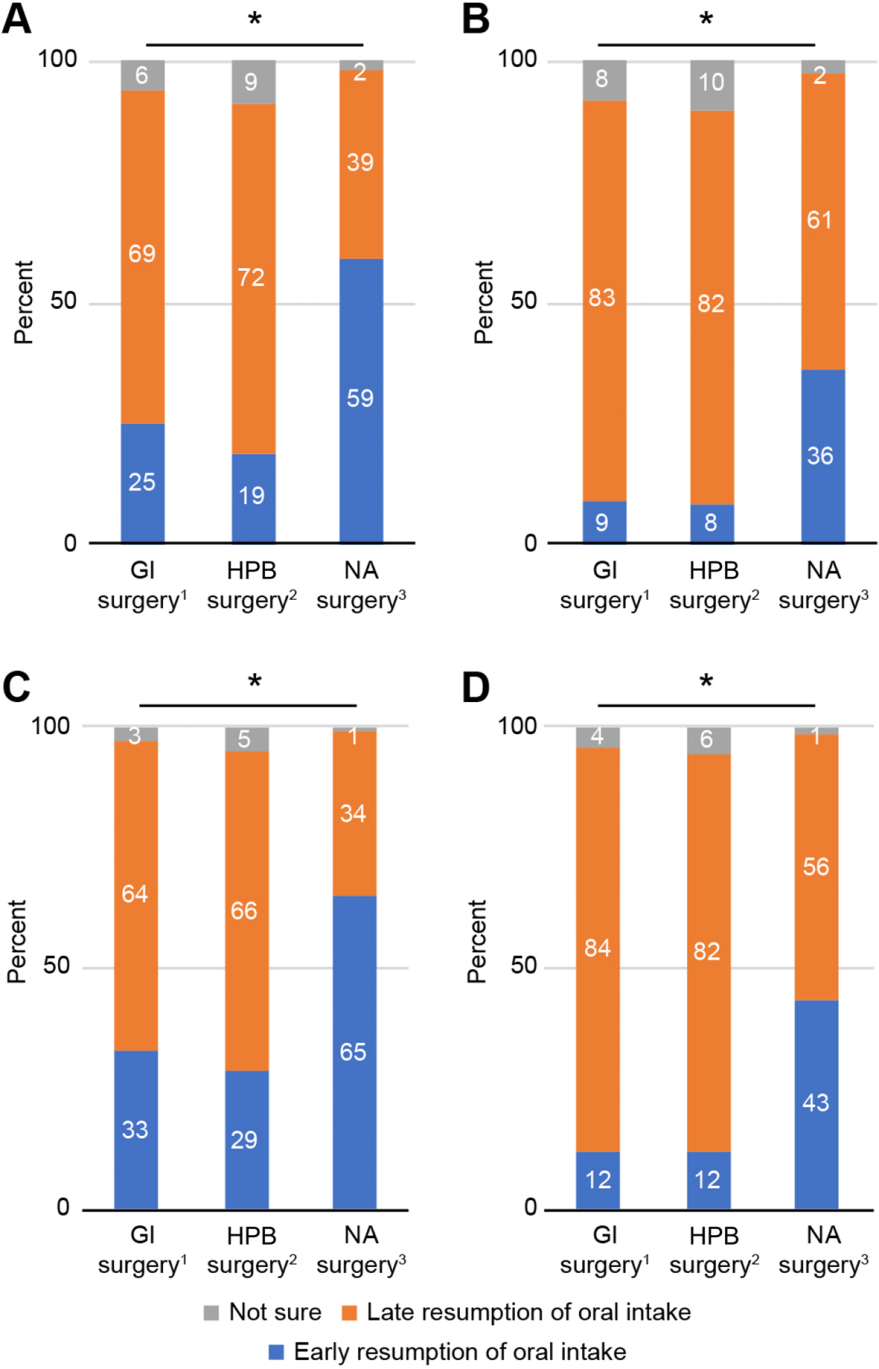
Divergence among the three surgery types for the postoperative resumption of oral fluids (**A** & **C**) and solid diet (**B** & **D**) in practice (**A** & **B**) and in perception (**C** & **D**). GI surgery, gastrointestinal surgery; HPB surgery, hepato-pancreato-biliary surgery; NA surgery, non-abdominal surgery. Data presented as % in columns. ^*,1-6^ Wilcoxon signed rank test, *P* < 0.001

This study focused on early oral intake; thus, for clear analysis, early resumption of postoperative oral intake was defined as diet given no more than 6 h postoperatively to patients, including the timepoints of “discharge from PACU” and “2-4 hours postoperation”, while late postoperative oral intake included all other timepoints except those who chose “not sure”. As shown in Fig. 4, despite the pattern similarity between gastrointestinal and hepato-pancreato-biliary surgery types, due to the sufficient number of responses, we found significant differences between each surgery group (*P* < 0.001). Regardless of practice or expectation, clinicians seemed to be more conservative regarding the resumption of oral intake after hepato-pancreato-biliary surgery, which may be a reflection of the complexity and heterogeneity of this large group of procedures, including pancreaticoduodenectomy. Obviously, the respondents were more receptive of early oral intake after non-abdominal surgeries. We also noticed that the practice and perception of respondents showed significant differences for each surgery group (*P* < 0.001). It is noteworthy that clinicians had more positive perceptions of early postoperative oral intake than reported in terms of practice in all three surgery types. After all, the surgical specialty objectively determined the resumption of oral intake, and the perceptions of the respondents also had an effect.

### Attitudes toward the early postoperative resumption of oral intake

**Fig. 5:**
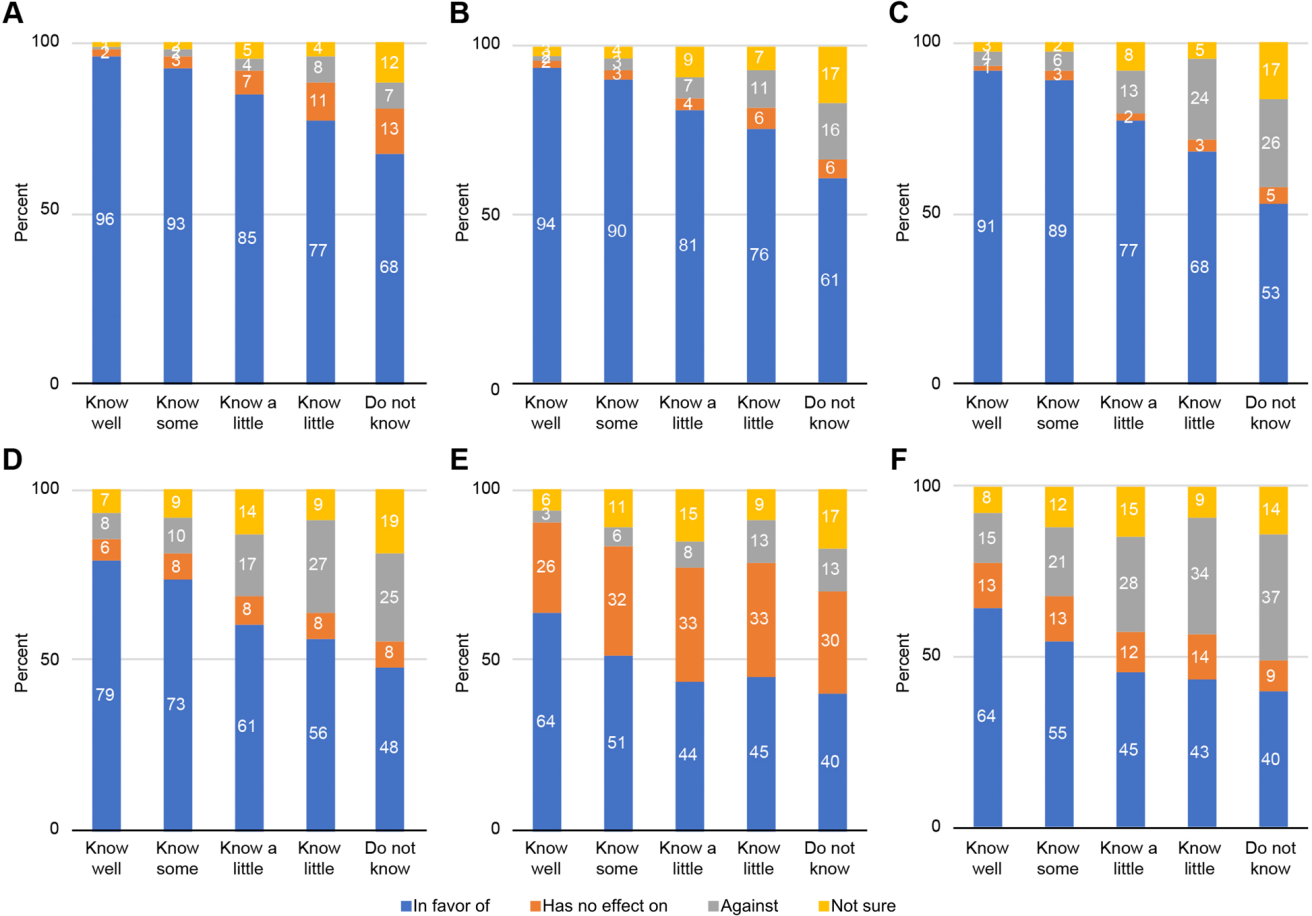
Association of attitudes toward the early resumption of oral intake and understanding of ERAS. **A** Improve patient satisfaction. **B** Improve overall prognosis. **C** Speed up bowel recovery. **D** Reduce postoperative complications. **E** Alleviate postoperative pain. **F** Alleviate postoperative nausea and vomiting. Data presented as % in columns

As shown in Fig. 5, for all the listed ERAS components, practitioners with higher level of understanding of ERAS were more likely to agree with the guidelines. Regardless of the level of understanding of ERAS, more than 55 % of the respondents agreed that early postoperative oral intake improves patient satisfaction and overall prognosis and speeds up bowel recovery. However, approximately one-third of practitioners believed that early oral intake after surgery had no effect on reducing postoperative pain. Meanwhile, respondents were more concerned that early oral intake was unfavorable for the alleviation of postoperative nausea and vomiting compared to other ERAS components, even those who claimed they “know well” the benefits of ERAS. As the level of understanding of ERAS increased, respondents reported more confidence in these benefits and less concerned in general.

### Understanding of ERAS affects the postoperative resumption of oral intake

As mentioned above, the respondents were divided into two groups: better understanding of ERAS and less understanding of ERAS. The differences in respondent characteristics are shown in Table 1. The group with a better understanding had more respondents from the Department of Anesthesiology, consisting of more males and attending physicians. Even though the majority of respondents had a university degree, more respondents in the better understanding group had an advanced degree. All of above differences were significant (*P* < 0.001).

**Table 1:** Characteristics of the respondents stratified by their understanding of ERAS

	Better Understanding of ERAS (*n* = 2954)	Less Understanding of ERAS (*n* = 2416)	*P* values
Department			
Anesthesiology	2726(92)	2054(85)	< 0.001*
Surgical Department	180(6)	257(11)	
Non-surgical Department	48(2)	105(4)	
Sex			
Male	1740(59)	1034(43)	< 0.001*
Female	1214 (41)	1382(57)	
Education			
College Degree	144(5)	318(13)	< 0.001*
University Degree	1792(61)	1755(73)	
Advanced Degree	1018 (34)	343(14)	
Title			
Attending	2321(79)	1300(54)	< 0.001*
Resident	399(14)	545(23)	
Nurse	228(8)	555(23)	
Other	6(0)	16(1)	

Data presented as n(%). ^*^ Chi-square test

**Fig. 6:**
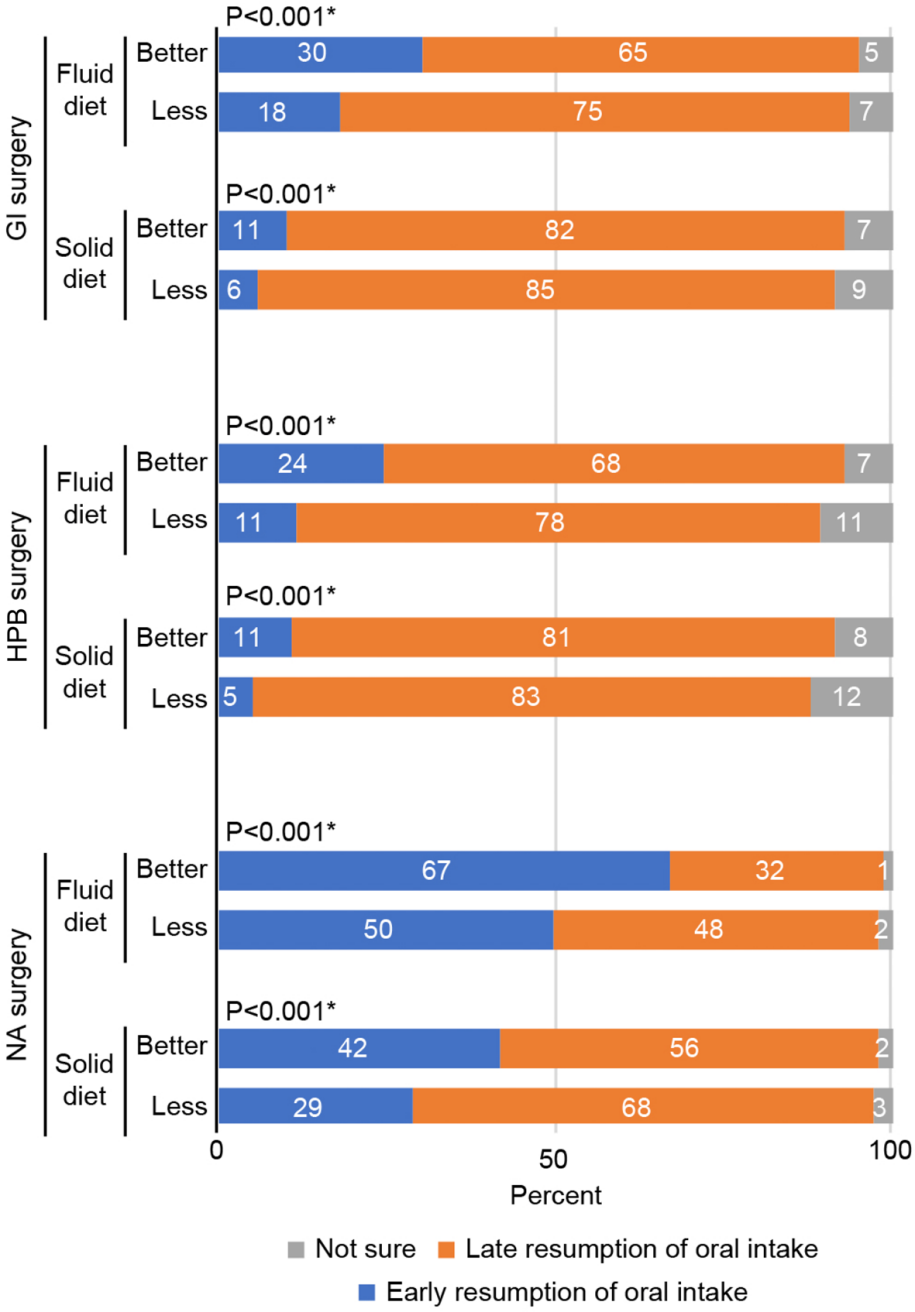
Understanding of ERAS affected the resumption of oral intake. GI surgery, gastrointestinal surgery; HPB surgery, hepato-pancreato-biliary surgery; NA surgery, non-abdominal surgery; Better = Better understanding of ERAS; Less = Less understanding of ERAS. Data presented as % in columns. ^*^ Chi-square test

Figure 6 shows how the understanding of ERAS affects decisions regarding the postoperative resumption of oral intake. In gastrointestinal surgery, 30 % of respondents in the better understanding group considered 6 h after surgery to be a sufficient period for a liquid diet, even though the less understanding group (18%) preferred conservative treatment. For hepato-pancreato-biliary surgery, we found more conservative answers in both groups, and the difference between the respondents in the better understanding group and those in the less understanding group was slightly larger in terms of early resumption of oral intake. However, after non-abdominal surgery, respondents in the two groups had the largest disagreement on the resumption of oral intake, with 67% percent of the respondents in the better understanding group preferring to resume oral fluid intake early versus only 50% of the respondents in the less understanding group. For the surgery groups, the pattern of early resumption of oral intake (blue blocks) looks like an upside-down ladder, from fluid to solid diet and from better to less understanding of ERAS, which indicates that a better understanding of ERAS leads to a more favorable choice for resuming oral intake early.

## Discussion

As this nationwide survey aimed to investigate the practice and attitudes towards early postoperative resumption of oral intake along with the understanding of ERAS, it was conducted to collect baseline data that would help establish a proper strategy for the widespread implementation of early postoperative oral intake in clinical practice. We found that early resumption of oral intake after non-abdominal surgery has been relatively well adopted. However, the implementation and conception of ERAS after gastrointestinal and hepato-pancreato-biliary surgery are discrepant, and the approach to recovery for these two types remains relatively conservative. In addition, a better understanding of ERAS would encourage practitioners to commence oral intake earlier in these patients. Therefore, consideration should be given to feasible solutions to the obstacles that may undermine the implementation of ERAS. Finally, a comprehensive and surgery-specific care protocol is urgently required to reduce variation and improve postoperative outcomes in China.

Postoperative resumption of oral intake is highly variable among gastrointestinal, hepato-pancreato-biliary and non-abdominal surgery. Indeed, after abdominal and intestinal surgery, due to the disruption of normal bowel motility, postoperative ileus may occur. For this reason, the timing of postoperative oral intake has long been debated [21, 22]. Traditional management usually includes remaining fasting until bowel sound or passage of flatus or stool, which are clinical parameters used to confirm resolution of ileus, and then commencing on clear fluids typically 2 to 5 days postoperation and finally progressing to a solid diet as tolerated [23]. However, these old-school parameters are dispensable evidence of resumed bowel function [24] and an early oral diet has been shown to be safe at 4 h after surgery in patients with nondiverted colorectal anastomosis [25]. It seems that gastrointestinal surgery is associated with a higher risk of postoperative ileus, so multimodal preventive strategies should be adopted to facilitate the recovery of gastrointestinal function if delayed gastric emptying or ileus occurs after surgery, including the use of nasogastric tubes prophylactically and remedially [7]. Moreover, different surgical methods may have different levels of risk, such as gastrointestinal surgery. Esophagojejunostomy is probably a more vulnerable reconstruction than distal or subtotal gastric resection, so patients undergoing the former may need a longer period of postoperative fasting [26]. Therefore, for gastrointestinal [6, 7, 27, 28] and hepato-pancreato-biliary [8, 9] surgeries, almost all ERAS programs recommend that patients should be allowed to resume a normal diet (may through enteric feeding tubes if needed [28])after surgery without restrictions according to their tolerance, even though none of them mention a specific number of hours or days postoperatively. To further explore early intake after surgery, more large-scale high-quality studies are needed. Our results confirm this clinical need.

After non-abdominal surgery, clinical practitioners had closer responses regarding the perception and practice of the resumption of oral intake, showing the best implementation and most confidence in ERAS. Indeed, for non-abdominal surgeries, most ERAS guidelines recommended that patients should be encouraged to resume oral liquids and a solid diet as soon as possible [10, 12–14], preferably within 24 h after surgery [11]. However, it’s worth noting that most literature cited by above guidelines were retrospective studies or data from intestinal surgery. A randomized controlled trial of early oral feeding in laryngectomized patients indicated the resumption of oral feeding on the first postoperative day is safe but little effect on reducing the length of hospital stay [29]. Namely, the return to normal food intake is essential component to resuming to normal activities, and more randomized controlled trials could be necessary to investigate the direct association of early oral intake with ERAS in non-abdominal surgeries.

Interestingly, most clinicians had more positive perceptions of ERAS than reported in terms of practice. We found that 4% of respondents considered that early oral intake after hepato-pancreato-biliary surgery is appropriate, although in practice, they were not sure of the most suitable course of action. Besides, most respondents agreed with the benefits of early oral intake; however, they still chose a conservative strategy in practice. More than half postponed the resumption of oral take due to concerns about choking cough (54%), postoperative nausea and vomiting (67%) and aspiration (73%). In thirty percentage of free text comments, respondents stated that they were concerned of a potential return to the operating room, as fed patients are at risk for aspiration. However, modern anesthetic techniques should mitigate the risk of aspiration in this uncommon scenario [30]. While a Cochrane systematic review [31] of colorectal surgery published in 2019 suggests that there is sufficient evidence to indicate that early enteral intake leads to a reduced postoperative length of hospital stay and risk of dying, all referred studies were of low quality. A lack of convincing data is the first stumbling block to ERAS implementation. However, the reality that scientific study does not always allow direct clinical correlation also cannot be ignored.

Among the three kinds of surgeries, respondents with a better understanding of ERAS guidelines were more likely to commence early oral intake after surgery. However, the numbers were not promising; our survey found that only 36% of respondents stated that they chose the timing of postoperative oral intake based on guidelines, while 55% chose the timing of postoperative oral intake based on clinical experience. This may be the reflection of the low consensus on the risk and significance of early postoperative oral intake. The current level of implementation of early oral intake in the hospital context is still low and based on type of surgery performed, the physician-patient relationship, and adherence to treatment [32]. Indeed, many other factors may influence clinical complication endpoints, such as the fitness of the patient, experience of the surgeon, resection sites, pain control, and success of the operation in resolving the underlying pathology [33, 34].

Reestablishment of oral intake as soon as possible after surgery has been incorporated into an increasing number of ERAS programs [4, 5]. The concept of ERAS involves a multidisciplinary team approach to solve the problems that cause complications and delay recovery by implementing evidence-based care protocols and changes in management through interactive and ongoing audits [35]. Our survey found that 22% practitioners considered that the timing of resumption of oral intake should be only decided and supervised by surgeons and nurses in the wards. But teamwork means engagement of all relevant stakeholders. Surgeons should break down entrenched surgical dogmas, and the collaboration of ward nurses is equally important for driving this program forward [36, 37]. As an anesthesiologist, our analgesic techniques should aim not only to provide optimal pain control but also to facilitate the tolerance of oral intake [38, 39] and to prevent postoperative nausea and vomiting using multimodal approaches [40, 41]. Nonetheless, since early postoperative intake does provide benefit, the identification and implementation of strategies to improve uptake of this ERAS component should be a priority.

As a first nationwide survey focused on the practice and attitudes towards early postoperative resumption of oral intake, there were also some limitations. First, this is a survey designed and conducted by anesthesiologists. Although respondents consisted of practitioners from anesthesiology, surgery and non-surgical departments, 89% of them are anesthesia related. Considering the feasibility of the study, we did not employ a strict probability sampling method during the study design, which may cause a population bias. Meanwhile, it’s clear that the ERAS guidelines are changing the general preconception of all perioperative clinical practitioners including surgeons, anesthesiologists and nurses, despite the population bias. Second, the survey or the questions were designed upon current clinical practice of mainland China, so the choices may not reflect current worldwide status of each question. Third, we did not collect the information about practitioners’ affiliations, nor did we differ or exclude the respondents who are majored in ambulatory surgery. Finally, all results were from perioperative health care practitioners’ answers, but not from the patients. As is known that patients may delayed their resumption of postoperative oral intake despite physicians’ arrangements.

## Conclusions

Different from preoperative fasting, practitioners still have great confusion and disagreement about postoperative resumption of oral intake. This study found out that the type of surgery is the key consideration that affects the practitioners’ decision to postoperative resumption of oral intake. Postoperative resumption of oral intake is highly variable among surgeries. Regardless of whether it is an abdominal surgery, the practitioners have a relatively consistent view on postoperative resumption of oral intake for the same type of surgery. Clinical practitioners had the best implementation and most confidence in early oral intake after non-abdominal surgery. This suggests that in the ERAS guidelines or consensus for different types of surgery, a clear schedule for postoperative resumption of oral intake could be given for patients without severe complications. In addition, A better understanding of ERAS would encourage practitioners to commence earlier oral intake. Future work should work on enhancing ERAS comprehension, formulating national or regional ERAS protocols and improving the implementation of ERAS, as will promote the overall postoperative recovery.

## Supplementary Information

**Additional file 1.**

**Additional file 2.**

**Additional file 3.**

**Additional file 4.**

## Data Availability

The datasets generated and analyzed during the current study are available from the corresponding author on reasonable request.
