# Supplementary material for: Level of ERAS understanding affects practitioners’ practice and perception of early postoperative resumption of oral intake: a nationwide survey
Source: BMC Anesthesiol. 2021 Nov 12;21:279. doi: 10.1186/s12871-021-01500-9 (PMC8588702; doi:10.1186/s12871-021-01500-9)
Supplement: Supplementary file 1 — Additional file 1. [file 12871_2021_1500_MOESM1_ESM.docx]

**Additional file 1. Online survey questionnaire**

1. What department are you affiliated with?

- Anesthesiology

- Surgical department

- Non-surgical department

1. What is your sex?

- Male

- Female

1. What is your education level?

- College degree

- University degree

- Advanced degree

1. What province do you reside in?
2. Please mark your current place of work.

- Tertiary hospital

- Community hospital

- Private hospital

1. Please indicate your title at work.

- Attending

- Resident

- Nurse

- Other (please specify)

1. Have you ever taken care of adult patients following surgery under general anesthesia?

- Yes

- No (survey will be terminated)

1. When do you allow your patients begin any fluid diet following surgery under general anesthesia? Please mark only one option for each surgery type.

|  | Discharge from PACU | 2-4 hours post operation | 6 hours post operation | Upon removal of nasogastric tube | Until passage of flatus | Until passage of feces | Without nausea, vomiting & distension | Not sure |
| --- | --- | --- | --- | --- | --- | --- | --- | --- |
| Gastrointestinal Surgery | ○ | ○ | ○ | ○ | ○ | ○ | ○ | ○ |
| Hepato-pancreato-biliary Surgery | ○ | ○ | ○ | ○ | ○ | ○ | ○ | ○ |
| Non-abdominal Surgery | ○ | ○ | ○ | ○ | ○ | ○ | ○ | ○ |

1. When do you allow your patients begin any solid diet following surgery under general anesthesia? Please mark only one option for each surgery type.

Same chart as question 8.

1. Reasons for your responses to questions 8 & 9. Please mark all that apply.

- Clinical experience

- Suggestion of anesthesiologists

- Recommendation of guidelines

- Regulation of hospitals

- Convenience of postoperative care

- Request of patients

- Others (please specify)

1. When do you think it is appropriate to begin a fluid diet following surgery under general anesthesia? Please mark only one option for each surgery type.

Same chart as question 8.

1. When do you think it is appropriate to begin a solid diet following surgery under general anesthesia? Please mark only one option for each surgery type.

Same chart as question 8.

1. What are your main concerns when deciding to delay the resumption of oral intake to later than 6 hours after surgery?

- Aspiration

- Distention, nausea & vomiting

- Choking cough

- Not fully awake from anesthesia

- Bowel obstruction

- Anastomotic leakage

- Wound infection

- Inconvenience of postoperative care

- Other (please specify)

1. What do you think is the impact of postoperative early oral feeding on the following clinical outcomes? (Options for all responses: In favor of/Has no effect on/Against/Not sure)

- Speed up bowel recovery

- Reduce postoperative complications

- Improve overall prognosis

- Improve patients' satisfaction

- Alleviate postoperative pain

- Alleviate postoperative nausea and vomiting (PONV)

1. What is your level of understanding about ERAS (Enhanced Recovery After Surgery)?

- Know well

- Know some

- Know a little

- Know little

- Do not know

1. How often do you apply ERAS?

- More than 80% of patients

- 40%~60% of patients

- 20%~40% of patients

- Less than 20% of patients

- Never

1. Do you know the ERAS recommendations regarding the initiation of postoperative oral feeding for your specialty?

- Without nausea and vomiting

- 4 hours after surgery

- 6 hours after surgery

- First 24 hours after surgery

- Postoperative Day 1 (POD1)

- No recommendations

- Not sure

- Other (please specify)

1. What is your specialty?
