# Supplementary material for: Level of ERAS understanding affects practitioners’ practice and perception of early postoperative resumption of oral intake: a nationwide survey
Source: BMC Anesthesiol. 2021 Nov 12;21:279. doi: 10.1186/s12871-021-01500-9 (PMC8588702; doi:10.1186/s12871-021-01500-9)
Supplement: Supplementary file 2 — Additional file 2. [file 12871_2021_1500_MOESM2_ESM.docx]

Supplemented Tabular information for figure 2

| Resumption of oral fluids after | Level of understanding of ERAS | Know well | Know some | Know a little | Know little | Do not know |
| --- | --- | --- | --- | --- | --- | --- |
| gastrointestinal surgery | Discharged from the PACU | 120 | 183 | 67 | 45 | 10 |
|  | 2-4 hours postoperation | 173 | 415 | 155 | 124 | 40 |
|  | 6 hours postoperation | 137 | 492 | 322 | 267 | 122 |
|  | Upon removal of nasogastric tube | 65 | 220 | 116 | 76 | 23 |
|  | Until passage of flatus | 154 | 550 | 318 | 248 | 130 |
|  | Until passage of feces | 7 | 23 | 17 | 14 | 7 |
|  | Without nausea, vomiting and distention | 76 | 188 | 81 | 50 | 26 |
|  | Not sure | 32 | 119 | 82 | 41 | 35 |
|  |  |  |  |  |  |  |
| hepato-pancreato-biliary surgery | Discharged from the PACU | 114 | 169 | 48 | 31 | 10 |
|  | 2-4 hours postoperation | 121 | 318 | 95 | 67 | 26 |
|  | 6 hours postoperation | 156 | 570 | 345 | 264 | 114 |
|  | Upon removal of nasogastric tube | 93 | 259 | 139 | 107 | 36 |
|  | Until passage of flatus | 124 | 472 | 258 | 240 | 106 |
|  | Until passage of feces | 6 | 24 | 14 | 17 | 6 |
|  | Without nausea, vomiting and distention | 103 | 210 | 126 | 73 | 30 |
|  | Not sure | 47 | 168 | 133 | 66 | 65 |
|  |  |  |  |  |  |  |
| non-abdominal surgery | Discharged from the PACU | 322 | 654 | 237 | 135 | 55 |
|  | 2-4 hours postoperation | 230 | 773 | 383 | 282 | 111 |
|  | 6 hours postoperation | 118 | 463 | 366 | 322 | 155 |
|  | Upon removal of nasogastric tube | 7 | 17 | 10 | 12 | 2 |
|  | Until passage of flatus | 29 | 73 | 54 | 47 | 30 |
|  | Until passage of feces | 1 | 3 | 0 | 5 | 1 |
|  | Without nausea, vomiting and distention | 49 | 171 | 81 | 53 | 20 |
|  | Not sure | 8 | 36 | 27 | 9 | 19 |
|  |  |  |  |  |  |  |
| Resumption of solid diet after | Level of understanding of ERAS | Know well | Know some | Know a little | Know little | Do not know |
| gastrointestinal surgery | Discharged from the PACU | 54 | 58 | 25 | 19 | 6 |
|  | 2-4 hours postoperation | 67 | 137 | 45 | 51 | 9 |
|  | 6 hours postoperation | 115 | 355 | 230 | 197 | 83 |
|  | Upon removal of nasogastric tube | 97 | 244 | 126 | 90 | 36 |
|  | Until passage of flatus | 291 | 926 | 487 | 346 | 163 |
|  | Until passage of feces | 38 | 142 | 80 | 67 | 27 |
|  | Without nausea, vomiting and distention | 60 | 154 | 56 | 40 | 21 |
|  | Not sure | 42 | 174 | 109 | 55 | 48 |
|  |  |  |  |  |  |  |
| hepato-pancreato-biliary surgery | Discharged from the PACU | 62 | 61 | 26 | 23 | 7 |
|  | 2-4 hours postoperation | 64 | 137 | 42 | 26 | 3 |
|  | 6 hours postoperation | 136 | 453 | 262 | 223 | 90 |
|  | Upon removal of nasogastric tube | 110 | 309 | 134 | 118 | 39 |
|  | Until passage of flatus | 227 | 728 | 389 | 285 | 138 |
|  | Until passage of feces | 32 | 98 | 57 | 46 | 17 |
|  | Without nausea, vomiting and distention | 83 | 206 | 98 | 77 | 30 |
|  | Not sure | 50 | 198 | 150 | 67 | 69 |
|  |  |  |  |  |  |  |
| non-abdominal surgery | Discharged from the PACU | 175 | 278 | 117 | 74 | 27 |
|  | 2-4 hours postoperation | 193 | 602 | 234 | 183 | 63 |
|  | 6 hours postoperation | 200 | 782 | 523 | 405 | 202 |
|  | Upon removal of nasogastric tube | 30 | 53 | 15 | 19 | 3 |
|  | Until passage of flatus | 67 | 208 | 138 | 100 | 42 |
|  | Until passage of feces | 6 | 20 | 11 | 6 | 3 |
|  | Without nausea, vomiting and distention | 80 | 202 | 87 | 64 | 33 |
|  | Not sure | 13 | 45 | 33 | 14 | 20 |
