# Supplementary material for: Level of ERAS understanding affects practitioners’ practice and perception of early postoperative resumption of oral intake: a nationwide survey
Source: BMC Anesthesiol. 2021 Nov 12;21:279. doi: 10.1186/s12871-021-01500-9 (PMC8588702; doi:10.1186/s12871-021-01500-9)
Supplement: Supplementary file 3 — Additional file 3. [file 12871_2021_1500_MOESM3_ESM.docx]

Supplemented Tabular information for figure 3

| Resumption of oral fluids after | Level of understanding of ERAS | Know well | Know some | Know a little | Know little | Do not know |
| --- | --- | --- | --- | --- | --- | --- |
| gastrointestinal surgery | Discharged from the PACU | 168 | 283 | 117 | 46 | 19 |
|  | 2-4 hours postoperation | 179 | 502 | 216 | 167 | 61 |
|  | 6 hours postoperation | 99 | 381 | 227 | 220 | 114 |
|  | Upon removal of nasogastric tube | 82 | 260 | 142 | 107 | 39 |
|  | Until passage of flatus | 116 | 456 | 262 | 209 | 93 |
|  | Until passage of feces | 6 | 26 | 15 | 21 | 9 |
|  | Without nausea, vomiting and distention | 96 | 221 | 135 | 75 | 36 |
|  | Not sure | 18 | 61 | 44 | 20 | 22 |
|  |  |  |  |  |  |  |
| hepato-pancreato-biliary surgery | Discharged from the PACU | 154 | 257 | 97 | 48 | 10 |
|  | 2-4 hours postoperation | 154 | 463 | 185 | 126 | 41 |
|  | 6 hours postoperation | 120 | 436 | 267 | 243 | 112 |
|  | Upon removal of nasogastric tube | 82 | 246 | 132 | 111 | 42 |
|  | Until passage of flatus | 120 | 374 | 218 | 191 | 87 |
|  | Until passage of feces | 8 | 34 | 17 | 17 | 10 |
|  | Without nausea, vomiting and distention | 103 | 291 | 162 | 94 | 44 |
|  | Not sure | 23 | 89 | 80 | 35 | 47 |
|  |  |  |  |  |  |  |
| non-abdominal surgery | Discharged from the PACU | 363 | 835 | 351 | 181 | 57 |
|  | 2-4 hours postoperation | 217 | 713 | 356 | 292 | 119 |
|  | 6 hours postoperation | 75 | 293 | 227 | 258 | 133 |
|  | Upon removal of nasogastric tube | 16 | 38 | 14 | 17 | 3 |
|  | Until passage of flatus | 31 | 92 | 59 | 53 | 25 |
|  | Until passage of feces | 2 | 4 | 6 | 1 | 2 |
|  | Without nausea, vomiting and distention | 56 | 200 | 119 | 57 | 39 |
|  | Not sure | 4 | 15 | 26 | 6 | 15 |
|  |  |  |  |  |  |  |
| Resumption of solid diet after | Level of understanding of ERAS | Know well | Know some | Know a little | Know little | Do not know |
| gastrointestinal surgery | Discharged from the PACU | 56 | 73 | 37 | 27 | 9 |
|  | 2-4 hours postoperation | 86 | 201 | 73 | 60 | 23 |
|  | 6 hours postoperation | 107 | 270 | 183 | 183 | 74 |
|  | Upon removal of nasogastric tube | 108 | 329 | 147 | 123 | 44 |
|  | Until passage of flatus | 267 | 877 | 454 | 305 | 153 |
|  | Until passage of feces | 30 | 141 | 99 | 72 | 30 |
|  | Without nausea, vomiting and distention | 83 | 205 | 106 | 73 | 26 |
|  | Not sure | 27 | 94 | 59 | 22 | 34 |
|  |  |  |  |  |  |  |
| hepato-pancreato-biliary surgery | Discharged from the PACU | 69 | 89 | 38 | 29 | 9 |
|  | 2-4 hours postoperation | 88 | 200 | 76 | 46 | 13 |
|  | 6 hours postoperation | 144 | 423 | 238 | 214 | 97 |
|  | Upon removal of nasogastric tube | 109 | 335 | 149 | 115 | 47 |
|  | Until passage of flatus | 195 | 644 | 357 | 285 | 129 |
|  | Until passage of feces | 25 | 98 | 54 | 46 | 17 |
|  | Without nausea, vomiting and distention | 102 | 290 | 159 | 94 | 33 |
|  | Not sure | 32 | 111 | 87 | 36 | 48 |
|  |  |  |  |  |  |  |
| non-abdominal surgery | Discharged from the PACU | 201 | 426 | 177 | 101 | 37 |
|  | 2-4 hours postoperation | 192 | 620 | 294 | 194 | 70 |
|  | 6 hours postoperation | 180 | 571 | 365 | 351 | 175 |
|  | Upon removal of nasogastric tube | 21 | 55 | 30 | 28 | 9 |
|  | Until passage of flatus | 64 | 219 | 113 | 106 | 45 |
|  | Until passage of feces | 8 | 17 | 13 | 8 | 4 |
|  | Without nausea, vomiting and distention | 90 | 264 | 138 | 73 | 42 |
|  | Not sure | 8 | 18 | 28 | 4 | 11 |
|  |  |  |  |  |  |  |
