# Supplementary material for: Level of ERAS understanding affects practitioners’ practice and perception of early postoperative resumption of oral intake: a nationwide survey
Source: BMC Anesthesiol. 2021 Nov 12;21:279. doi: 10.1186/s12871-021-01500-9 (PMC8588702; doi:10.1186/s12871-021-01500-9)
Supplement: Supplementary file 4 — Additional file 4. [file 12871_2021_1500_MOESM4_ESM.docx]

Supplemented Tabular information for figure 5

|  | Level of understanding of ERAS | Know well | Know some | Know a little | Know little | Do not know |
| --- | --- | --- | --- | --- | --- | --- |
| Improve patient satisfaction | In favor of | 731 | 2028 | 982 | 669 | 266 |
|  | Has no effect on | 19 | 76 | 78 | 96 | 51 |
|  | Against | 5 | 41 | 42 | 67 | 29 |
|  | Not sure | 9 | 45 | 56 | 33 | 47 |
|  |  |  |  |  |  |  |
| Improve overall prognosis | In favor of | 716 | 1975 | 934 | 654 | 238 |
|  | Has no effect on | 13 | 62 | 44 | 50 | 24 |
|  | Against | 14 | 67 | 76 | 99 | 64 |
|  | Not sure | 21 | 86 | 104 | 62 | 67 |
|  |  |  |  |  |  |  |
| Speed up bowel recovery | In favor of | 699 | 1950 | 893 | 589 | 208 |
|  | Has no effect on | 11 | 55 | 24 | 29 | 18 |
|  | Against | 34 | 131 | 147 | 204 | 101 |
|  | Not sure | 20 | 54 | 94 | 43 | 66 |
|  |  |  |  |  |  |  |
| Reduce postoperative complications. | In favor of | 606 | 1605 | 701 | 487 | 189 |
|  | Has no effect on | 46 | 176 | 98 | 67 | 30 |
|  | Against | 60 | 221 | 202 | 231 | 99 |
|  | Not sure | 52 | 188 | 157 | 80 | 75 |
|  |  |  |  |  |  |  |
| Alleviate postoperative pain. | In favor of | 489 | 1121 | 505 | 392 | 158 |
|  | Has no effect on | 201 | 703 | 385 | 284 | 118 |
|  | Against | 26 | 123 | 93 | 109 | 50 |
|  | Not sure | 48 | 243 | 175 | 80 | 67 |
|  |  |  |  |  |  |  |
| Alleviate postoperative nausea and vomiting | In favor of | 490 | 1197 | 525 | 374 | 156 |
|  | Has no effect on | 103 | 283 | 136 | 118 | 37 |
|  | Against | 111 | 451 | 327 | 293 | 144 |
|  | Not sure | 60 | 259 | 170 | 80 | 56 |
